# A Treatise on the Venereal Diseases of the Eye

**Published:** 1832

**Authors:** 


Art. VIII. *A Treatise on the Venereal Diseases of the Eye.* By Wm. Lawrence, F. R. S., late Professor of Surgery to the Royal College of Surgeons in London, Surgeon to St. Bartholomew's Hospital, &c., &c. London: 1830. pp. 337.

When we recollect how many tissues enter into the composition of the eye and its appendages, we cannot wonder at the variety of its diseases. It was not till since the middle of the last century, that these maladies were studied in the true spirit of medical philosophy, especially in Great Britain and the United States. Even down to the commencement of the present century, no works of unquestionable merits on the diseases of this organ, had appeared elsewhere than in Germany, France, and Italy. At present, and indeed for the last thirty years, the surgeons of London seem to have regarded these maladies as entitled to attention, and they have consequently been redeemed from the keeping of professed oculists—most of whom were mere pretenders. Among those who have latterly dedicated themselves to this interesting but neglected branch of the healing art, we may enumerate the highly respectable names of Hey, Ware, Saunders, Vetch, Adams, Wardrop Travers, Guthrie, and Lawrence; to the last of whom we are indebted for the work of which we now propose to give some account.

As it is one of the latest, so it is one of the most authoritative books, on the subject of which it treats; for the opportunities of the Author have been ample, while his zeal and talents are known to be of the highest order. According to the title of his treatise, he limits himself to those diseases of the eye which are of venereal origin, dividing them into two species—*Gonorrhœal* and *Syphilitic*, the former chiefly seated in the Conjunctiva, the latter in the Iris.

The existence of gonorrhœal ophthalmia has, sometimes, been questioned; but had any doubts existed with us, they would have been removed by the collection of cases which Mr. Lawrence has appended to this part of his work. They amount to 24, and seem to us collectively to prove, that the mucous membrane of the eye is susceptible of being acted on by the gonorrhœal virus; or at least, of sympathizing with the mucous lining of the urethra.

Our Author begins by dividing gonorrhœal inflammation of the eye into three forms:—1st. Acute inflammation of the conjunctiva;—2d. Mild inflammation of that membrane;—and 3d. Inflammation of the sclerotic coat, sometimes extending to the iris.

The first variety offers the characters of intense purulent ophthalmia, from a common cause; excessive fulness of the vessels of the conjunctiva, with great redness; a swelling of that membrane throughout its whole extent; the tumefaction of that portion which covers the sclerotic coat frequently becoming so great, as to produce a high degree of chemosis; a feeling, as in common ophthalmia, of dirt or sand in the eye; considerable intolerance of light; and a profuse yellow puriform discharge. The inflammation soon extends to the cornea; the whole globe of the eye, the orbit, and indeed the head, are seized with agonizing pain; the intolerance of light rapidly increases; and inflammatory fever, more or less intense, is awakened.

Now, all these symptoms are frequently met with in conjunctivitis from other causes. How then are we to establish the diagnosis of the gonorrhœal variety? Mr. Lawrence is aware of this difficulty, and admits that the local symptoms are not sufficient for this purpose. We must inquire into the previous or present existence of a gonorrhœa. If the patient had been, and still was, free from that malady, the presumption would be against a venereal taint of the eye. Too much reliance, however, should not be placed on this observation. We have met with one case, at least, which seemed to show, that an individual, after inoculating his own eye with the gonorrhœal virus, may communicate it to others who wash in the same basin, or use the same towel.

In this manner, we once knew a disease which had the characters of gonorrhœal ophthalmia, communicated by a journeyman shoemaker, to several others who worked and lodged with him. Purulent ophthalmia, when not produced by accidental violence, is well known generally to affect both eyes; but the gonorrhœal disease, Mr. L. thinks, for the most part, attacks one eye only, though it sometimes extends to the second. The former commonly begins in the mucous membrane of the eye-lids, while the latter, in one case, the *commencement* of which our author witnessed, began in that part of the membrane which overspreads the eye-ball. This, however, is but a single observation; and in the midst of so much uncertainty, he is obliged to have recourse to the *degree*, instead of the *kind* of symptoms, a method of distinction, on which little reliance can be placed. In comparing the two affections, in this respect, he has found the gonorrhœal to be more intense, rapid and destructive in its progress, than that from common causes. From this rule we must, however, except those inflammations which are the effect of direct external violence. Thus, we have seen the explosion of a fulminating cracker, near one of the eyes, produce all the symptoms of the most intense and destructive gonorrhœal ophthalmia, including chemosis and total sloughing of the cornea, with ultimate escape of the lens.

In a country, like our own, where purulent ophthalmia is an endemic, many cases closely resembling the gonorrhœal, present themselves, and give the medical adviser no little perplexity. Gonorrhœa, however, is, in the Western states, a much rarer disease, than in Europe, if we may credit Mr. Hunter, who says that most men have one time or other in their lives labored under that malady. An immense majority, therefore, of those who are attacked with purulent conjunctivitis, may be presumed free from gonorrhœal contamination. When others, inhabiting the same locality are affected with the same disease, the presumption will be against a venereal origin. Finally, if the attack occur between the summer and the winter solstice, it will be correct, in the absence of the proofs of a venereal taint, to ascribe the disease to miasmata, or the cause, whatever it may be, of endemic ophthalmia.

The effects of gonorrhœal inflammation of the eye, when it is not promptly subdued, are most disastrous. The immediate læsions are ulceration, or sloughing of the cornea, suppurations in the chambers of the eye, and extensive interstitial deposites. As consequences of these injuries of structure, we have, in different cases, what the people, not inappropriately, call *bursting* of the eye-ball. An opening being made through the cornea, or that membrane, in whole or in part thrown off, the aqueous and sometimes the vitreous humor escapes. The anterior segment or the whole globe collapses. Or in milder cases, the iris protrudes through an aperture, the pupil is obliterated, and the remaining cornea, rendered opake, adheres permanently to the iris; læsions, which of course produce irremediable and permanent blindness. In other cases, accumulations behind the latter membrane, push it forward, against the cornea, which becomes more conical, is changed in its structure, and becomes staphylomatous.

Mr. Lawrence discusses the question, whether an individual laboring under gonorrhœa can infect his own eyes with virus from his urethra. The *onus* of authority seems to incline to the negative; but Mr. Wardrop communicated to our author two cases, which are clearly in the affirmative. First, a young gentleman laboring under gonorrhœa, inadvertently touched his eyes when his fingers were contaminated with the discharge:—the contact was followed by violent puriform ophthalmia, and ended in the suppuration and collapse of the eye-balls. Secondly, a soldier who had gonorrhœa, was advised to bathe his eyes in his own urine, as a remedy for a slight affection of the lids: purulent ophthalmia seized one eye, which suppurated and burst.

The question whether the gonorrhœal discharge from one person can produce ophthalmia in another, is, in the opinion of our Author, decided in the affirmative, by a conclusive, if not ample experience. Such cases, of course, cannot be very numerous; and Mr. L. acknowledges that he has not met with any. Mr. Wardrop has, however, furnished him with two; and as they come from such respectable authority, we shall notice one of them. An old lady, being in the chamber occupied by her son, who labored under gonorrhœa, wiped her face with a towel which he had contaminated with the discharge from his urethra. Purulent ophthalmia immediately ensued, and, in a few days destroyed one of her eyes. Delpech and Bacot have published other cases, equally in point.

It must be admitted, however, that in most cases of what is called gonorrhœal ophthalmia, the disease cannot be traced up, to the application of the discharge from the urethra. Can purulent ophthalmia, when it occurs in one laboring under gonorrhœa, result from that malady, independent of the deposit of the poison upon the conjunctiva? It must be admitted that a great majority of gonorrhœal patients escape ophthalmia; and still further, that the supervention of the latter upon the former, is not a proof of the relation of cause and effect; for a man affected with gonorrhœa, is obnoxious to all the common causes of ophthalmia. If, however, such supervention occur, without any obvious cause, the presumption certainly is, that the affection of the conjunctiva is produced by that of the urethra. But in what manner, if not by inoculation? We know of none, but sympathy. Our author refers to the extension of rheumatic inflammation from one group of fibrous membranes to another; although there is no continuity of structure. It should be recollected, however, that rheumatism is the offspring of causes that in general act on the whole system, while gonorrhœa is always the effect of a local impression. Still, there are analogies, perhaps, for the supposed sympathy of the mucous membrane of the eye with that of the urethra.

Many writers have asserted, that as the inflammation of the eye arises, that of the urethra ceases; they have, therefore, denominated it metastasis, and advised the reproduction of the latter as a means of curing the former. Such are the opinions and advice of Scarpa, Beer, and Richter; but our author has not witnessed the cessation of gonorrhœa upon the occurrence of conjunctivitis, though it has generally, not always, been mitigated. It is easy to understand, that the primary disease, in some cases, may cease altogether, in others be only mitigated—

on the access of the secondary; for pathology presents us with examples of this kind.

The treatment of gonorrhœal ophthalmia requires great promptness and energy. Procrastination or timidity in practice, is almost inevitably followed by sloughing or ulceration of the cornea, and the total destruction of sight. The proper treatment is, of course, the antiphlogistic. Copious venesection, with subsequent cupping or leeching, is indispensable; and must be repeated at short intervals, till the congestion and pain are removed. Our readers will be surprised, perhaps, to learn, that few diseases, seated even in the great viscera of organic life, require for their cure so great an abstraction of blood; not that the life of the patient, but his vision, is in jeopardy. Mr. Wardrop, informed our Author, that in one case, he had to draw 170 ounces in a few days, the patient, moreover, being a young female. The event was favorable.

We are somewhat surprised, that our Author says little or nothing in favor of nauseants and cathartics, certainly most efficient antiphlogistics, and well calculated, not only to diminish the energy of the circulation in, but to divert from the head. We find it somewhat difficult to subscribe to the propriety of such abstractions of blood, as ten pints and a half from a girl, in a few days; and with much deference to the high authority of Mr. Wardrop, we incline to the opinion, that a liberal use of tartar emetic and opium, would have rendered half that quantity sufficient. There are, certainly, other remedies for inflammation, than the loss of blood, although, beyond a doubt, that is the principal. This fact is recognised by those who advise the administration of calomel, in the malady now under consideration. Our Author thinks lightly of this practice, and fortifies his own experience with that of Beer and Delspech; others, however, have thought it beneficial, and we have certainly, seen violent ophthalmias from other causes, give way to a speedily induced mercurial action. Of the advantage of combining opium with mercury, or antimony, after great depletion, we feel quite assured. The extreme irritability of the general system, consequent upon copious bloodletting, requires a narcotic, and in most cases, the abatement of this irritability is followed by an abatement of the inflammation.

If the cornea should have already sloughed off, this active antiphlogistic course is useless, and may even be prejudicial if persisted in. A circumscribed ulceration does not, however, contra-indicate the use of antiphlogistics; but, on the contrary, they often contribute to arrest its spread, and thus preserve the vision.

Our author does not ascribe much efficacy to blisters, and they must never be employed till the inflammation begins to abate. They should be applied to the nucha, and kept running by an irritating cerate.

Topical applications are not to be neglected. Mr. L. has employed them chiefly to mitigate the sufferings of the patient, which are often intense. Sometimes cold, at other times warm, lotions have been found the best. A solution of acetate of lead, in rose water or a decoction of poppy heads, has been found beneficial. The sulphate of zinc and other astringents, sometimes do good; especially when the powers of the general system are weak, and the ulceration of the cornea spreads with ragged edges and a whitish or dull yellow color. In these cases a solution of alum is often serviceable.

Mr. Lawrence informs us, that Mr. Melin has employed a strong solution of nitrate of silver, at the commencement of the disease, for the purpose of cutting it short. Mr. Bacot and Dr. Ridgeway, have pursued the same practice, with decided success, both in gonorrhœal and common conjunctival inflammation. They used a solution of ten grains to the ounce of distilled water. Dr. O'Halaran bears ample testimony, to the good effects of the same treatment. In addition to the solution of nitrate of silver, he touched the inside of the everted eyelids with a crystal of the sulphate of copper. We are enabled, from experience, to speak well of this practice in every stage of common purulent ophthalmia; a disease in which we have, indeed, found these medicines, in a concentrated state, and other active applications, of far more service than mild collyria.

Incisions into, or the complete excision of the chemosis, have been recommended, but our author does not seem disposed to sanction the practice, nor have we, in common conjunctivitis, seen it of much avail.

Dismissing the subject of acute gonorrhœal ophthalmia, we come to our author's second species, mild gonorrhœal inflammation of the conjunctiva; characterized by increased mucous secretions and external redness of a bright scarlet tint, arising from distension of the superficial vessels of the globe. We need not dwell on this variety. When the patient is of a full habit, active measures are necessary; but, in general, we can succeed with astringents, of which the solution of nitrate of silver is most convenient and efficacious.

The last variety of gonorrhœal ophthalmia, is complicated with sclerotic and iritic inflammation. The vessels lying behind the conjunctiva, over the white of the eye, are distended; and the whole of this zone of the sclerotic membrane, assumes a pink or purplish red color. The conjunctiva is but little affected; the flow of tears is copious; the pain and sense of tension in the globe are great; and the impress of a bright light is intolerable. The inflammation extending to the iris, deprives that membrane of its brilliancy, and causes it to assume a dull and deeper hue. The pupil contracts, and lymph is effused and deposited on its margin. The conjunctiva at length becomes loaded with blood, and the cornea suffers in its transparency.

This variety must be treated by the loss of blood, both general and local, and by other antiphlogistic means. Warm fomentations are generally agreeable to the patient. Seclusion from light is absolutely necessary, as long as the patient feels its admission painful. Blisters frequently do good. Colchicum has often been used with advantage. In many of these cases, the sclerotica has indeed a rheumatic disposition. It may seem strange to some of our readers, to find rheumatism, ophthalmia, and gonorrhœa associated. Such nevertheless is the fact. Our Author has met with this combination, and quotes Mr. Brodie for the same fact. We, ourselves, have seen rheumatic pains excited by gonorrhœa; and recollect one case in which the latter malady produced rheumatism, which, with terrible perseverance, attacked most of the large joints, in succession, and did not entirely leave the patient for years. It is not easy, perhaps, to account for these sympathies of the fibrous structure, either ophthalmic or articular, with the mucous membrane of the urethra. Mr. Lawrence supposes that idiosyncracy is concerned in their production, for he has seen them several times in the same individual; but this is the mere expression of a fact, not its rationale.

To the account of *gonorrhœal* ophthalmia, of which we have given a condensed but faithful view, Mr. L. has appended, as we have already stated, the detailed histories of a great number of cases. Many of them are instructive, and taken together, they serve to exhibit the symptoms, and illustrate the treatment of all the varieties of this malady, but our limits do not admit of a further notice of them; and we shall proceed to an analysis of the second part, devoted to the deeply important subject of *syphilitic* diseases of the eye.

These are divided into two varieties—first, *Syphilitic Iritis*; secondly, *Syphilitic Ulceration of the Eye-lids*. We shall take them up in the order in which they stand.

The cavity which is imperfectly separated into two compartments by the floating iris, is lined with a serous membrane, which exhales and absorbs the aqueous humor of the eye. In iritis this membrane, as well as the iris itself, is inflamed. The legitimate and common product of this inflammation is coagulable lymph. The deposition of this effusion upon the iris, and in its pupillary aperture, changes the color of that part, at first renders its motion sluggish, then terminates them altogether, agglutinates the organ to the cornea or the capsule of the lens, and plugs up the pupil or effects its permanent contraction. Iritis, then is an adhesive inflammation of a serous membrane. This malady is of course attended with distension of the vessels of the sclerotica, imparting redness to the adnata of the eyes, with increased sensibility to light, and a flow of tears.

The change of color which the iris suffers is remarkable. In most cases the tint is dull and muddy. If the iris were red colored, the hue which it assumes is yellowish or greenish, while a blue eye will sometimes be altered to a bright green. A dark colored iris becomes in most cases reddish. In all cases its natural briliancy is lost, and its beautiful fibrous structure nearly or quite disappears.

The deposition of lymph occurs under various modifications in Iritis. 1st. Into the texture of the iris, when it produces the changes of color just described. 2d. In a thin layer on the surface of that membrane. 3d. In distinct masses from the size of a pin's head to a split pea, on various parts of the iris. 4th. When the inflammatory action is violent, blood itself is sometimes effused, and intermixed with coagulable lymph. 5th. A mass of lymph sometimes fills the pupil, or agglutinates its margin, partially or generally, to the capsule of the lens. 6th. Lymph may be effused in such quantities into the posterior chamber, as to cause a bulging of the sclerotica, or to press the iris forward against the cornea. Cases of this kind, have been called suppuration of the eye-ball; but our Author believes that the inflammation, in Syphilitic Iritis, however violent, is always adhesive, and consequently that suppuration never takes.

We have already said, that the motions of the iris are seriously impaired, by the disease now under consideration. In reference to its diagnosis, this fact must not be forgotten. In most cases this loss of motion finally becomes perfect, with an exceedingly contracted pupil. This aperture, moreover, frequently loses its circular figure, and is often drawn from its formal situation, towards the root of the nose.

The redness of the sclerotica, of which we have spoken, is generally greatest in a zone near the cornea, gradually shaded off behind. This redness is that of the pink, while the hue which the super-incumbent vessels of the conjunctiva presents, is scarlet. When the iritis is intense, the redness is fiery. This flame-colored zone lasts as long as the iritis continues, and ceases with it.

The cornea does not assume the red color of the sclerotica, but becomes hazy, at length nebulous, opake, and sometimes ulcerated. The aqueous humor appears to preserve its transparency.

In syphilitic iritis, the sufferings of the patient are chiefly at night. After a day of comparative ease, with the approach of evening, or at bed time, the pain, which is usually seated in the brow, becomes intense, so as often to prevent sleep till towards morning. In this characteristic, syphilitic ophthalmia differs in no respect from the endemic ophthalmia of the West, which, as to pain, always suffers a nocturnal exascerbation.

Many cases of Syphilitic Iritis excite but little constitutional disturbance; but when the disease is really acute, it may awaken severe fever, with restlessness, headache, and insomnolency—the pulse being full and tense, the tongue white, the appetite suspended, and the bowels costive.

If not arrested, Syphilitic Iritis may extend itself to the choroid and retina, producing amaurosis, with irremediable blindness; a result which should be recollected, when we are consulted on the subject of artificial pupil. It is, indeed, this extension of inflammation to the posterior tunics of the eye, that is most to be dreaded.

Mr. Lawrence thinks that Syphilitic Iritis, may be distinguished from that occasioned by other causes, by the tubercular depositions of lymph; the reddish brown discoloration of the iris on its inner circle; the nocturnal exascerbations of pain, assimilating it to other syphilitic affections; the angular disfiguration of the pupil, and its displacement towards the root of the nose; together with the previous or concomitant occurrence of syphilitic symptoms. In iritis, from gout, or common causes, the pupil generally retains its normal figure and position.

Iritis is one of the sympathetic or secondary affections in syphilis. We are ignorant of the manner in which it is produced, as we are, of the production of nodes, ulceration of the throat, and cutaneous discolorations. It is among the earliest of the secondary symptoms, and therefore sometimes occurs before the primary chancre is cured.

Our author thinks that Syphilitic Iritis is not often infantile. He has seen but two cases, in which children seemed to have derived it from their parents.

It has sometimes been thought, that the iritis which occurs in syphilis, is caused by the mercury administered for the cure of that disease; but Mr. L. in his extensive experience, has met with no fact which goes to sustain this opinion. Iritis has often occurred in syphilitic patients, treated on the antiphlogistic plan without mercury.

Taken early, Syphilitic Iritis may generally be brought to a favorable termination; and whatever may be the ravages of the disease, as long as they are confined to the iris, the case is not desperate; for extensive deposites of coagulable lymph may be absorbed, and an artificial made to supply the place of the natural opening; but if the eye have become amaurotic, all of course is lost. Mr. L. thinks the case hopeless, when the whole iris presents a change of color, with considerable contraction of the pupil, and an opake substance in it; with intense redness externally; acute and deep seated pain, and total extinction of sight.

*The treatment* of Syphilitic Iritis, requires, 1st., a resort to antiphlogistics; 2d., the administration of mercury; 3d., the application of belladonna or some other narcotic, capable of dilating the pupil.

When the symptoms are acute, and especially when the inflammation is extending itself to the posterior tunics of the eye, no time should be lost in resorting to an active antiphlogistic treatment. Copious bloodletting, both general and local, to be frequently repeated; active purging; antimonials; abstinence, and an exclusion of light, are indispensable. In milder cases, a treatment less energetic, may suffice. Topical applications are of but little service. Tepid water, a decoction of poppy heads, or, if agreeable to the patient, cold water, will be most soothing. Blisters have not done much good, and add to the excitement if employed too early.

Although antiphlogistic measures are indispensable, in most cases of Syphilitic Iritis, and of themselves frequently effect a cure, still we must not depend solely on them. Mr. Lawrence has frequently seen the morbid action in the capillary vessels, continue after large and repeated bleedings, the effusions of lymph still going on, and alterations of structure progressively developing themselves. In many cases, then, something more than antiphlogistics is required, and this *desideratum* we have supplied to us in mercury.

Some oculists have proposed to treat the disease entirely with this medicine; but this practice is both unphilosophical and unsuccessful. It can only succeed in mild cases, and, even in these, will be more successful if preceded by evacuants. It should, therefore, rarely be made the first remedy in the order of time; and, quite as rarely, be omitted, as a second. The best mercurial preparation is calomel; and to be successful, it should be administered in large and salivating doses. Our author speaks of two, three or four grains, with one-fourth, one third, or half a grain of opium, every eight, six, or in urgent cases, every four hours. But in this country, this would scarcely be regarded, as an energetic mercurial course; and as the object is to induce a speedy and copious salivation, we see no reason, why a more liberal administration should not be made. It might, certainly, in desperate cases, be exhibited in portions, three times as great. On the establishment of a mercurial action, the remaining iritic inflammation rapidly abates; and in most cases, a salutary absorption of the effused lymph, as speedily occurs; at the same time, the redness of the eye disappears, and the pain ceases. In all this, there is nothing incredible. The disease is syphilitic inflammation of a *serous* surface. Now, it is well known, that mercury exercises great power over serous inflammation from any cause; while it is universally acknowledged to control syphilitic actions beyond any other means; hence, no other malady presents greater aptitudes for this medicine, than that other consideration .In many cases the remedy may be laid aside, as soon as the salivation commences; but our Author has seen others, in which a gentle ptyalism, had to be kept up for several weeks. In mild cases, the blue pill, or the *hydrargyrum cum creta*, will be sufficient.

The third part of the treatment, consists of the means which prevent contraction or closure of the pupil, while the cure of the inflammation is proceeding. Without a resort to these, the pupil would often be obliterated before the inflammation could be subdued, even by the most energetic treatment. For this purpose, the extract of belladonna, stramonium, or hyoscyamus, may be employed. A scruple should be rubbed down with an ounce of distilled or rain water, and filtered, before it is dropped into the eye. Or the extract, brought to the consistence of honey, may be rubbed on the brow. The ophthalmic surgeons of Germany, have recently employed, for the same purpose, the alkaline principles of belladonna and hyoscyamus, under the names of atropia and hyoscyamine. Dr. Reisinger speaks in high terms of the latter. It dilates the pupil far more extensively than either of the extracts, excites neither pain nor redness, and does not weaken the sight, however long it may be applied. One grain of the hyoscyamine may be dissolved in a drachm of distilled water, and a single drop will generally produce the desired effect. Whatever narcotic we may select, it should be applied sufficiently often, to keep the pupil constantly dilated. When the inflammation is still violent, it is difficult to effect much dilitation; but the medicine does no harm, especially if it be rubbed about the eye, and should not be omitted. In subsequent stages, when adhesions have taken place between the iris and the capsule of the lens, they are sometimes stretched by the action of the narcotic; and have even been ruptured, so as to liberate the organ; an effect which could not readily be produced by any other means.

Such is the ordinary treatment of Syphilitic Iritis; a method founded on sound therapeutic views, and sanctioned by ample experience. It does not, however, succeed in all cases. Certain constitutions do not bear bloodletting; others become irritable under the use of calomel; and others are worn out. In short, cases will arise which require a different treatment. To this end, Dr. Hugh Carmichael has lately recommended the internal use of the oil of turpentine. He rubs it into an emulsion, with yolk of an egg and sweet almonds. Before, or in connexion with this medicine, more or less depletion will be necessary. Mr. Carmichael, in several well marked cases of syphilitic iritis, has seen the pain and redness quickly removed, effused lymph absorbed, and vision restored. Mr. L. has not used it.

We shall state, in concluding this subject, that iritis from other causes, is successfully treated by the same remedies, which cure that from syphilis; a remark which would have been equally applicable to common and gonorrhœal ophthalmia.

The appendix to that part of Mr. Lawrence's book, which we have just analysed, contains the histories of 29 cases. They are full of instructive matter, but we cannot notice them further.

The 6th and last chapter is devoted to syphilitic ulceration of the eye-lids, an affection not formally noticed in the common treatises on syphilis, and ophthalmic diseases. It may exist, either with or without other secondary symptoms. In some cases, Mr. Lawrence has not been able to obtain from the patient an acknowledgement of the previous existence of chancre. Perhaps, in fact, it had not existed. May not the virus sometimes be accidentally applied to the eye-lids, and produce in that part an original chancre?

Our Author has seen the disease exhibit both an acute and chronic character. When acute, the ulcer is phagedenic, with a red margin, sharp edges and foul unequal surface, on which bloody points are seen, the pain is severe, and the eye-lid rapidly destroyed. In the chronic variety, there is swelling and some hardness of the sore, with expansion of the cutaneous texture, instead of loss of substance, and little or no pain.

In some cases the ulcer is seated in the skin, in others it affects the conjunctiva only, in others it commences on the ciliary margin, and descending or ascending, involves all the tissues.

Mr. L. thinks that no other ulceration of the eye-lid, can be mistaken for the syphilitic. From the cancerous, it differs, in not being preceded by a scirrhous tubercle, in forming and advancing more rapidly, in extending most commonly to all the tissues, while cancer affects the cutaneous only, and in being associated with syphilitic symptoms.

The treatment of syphilitic ulceration of the eye-lid is decidedly mercurial. As soon as the system feels the effect of the internal use of mercury, the sore loses its syphilic character, and quickly heals. Befcre our Author came to rely on this remedy alone, he tried the antiphlogistic and emollient plans, but found them utterly unavailing. He very justly remarks, that the deformity and inconvenience, resulting from the loss of an eye-lid, are so great, that sarsaparilla and other inferior means should never be trusted, when the preparations of mercury present so certain a resource.

We have thus laid before our readers a plain and ample account of Mr. Lawrence's book, a work which we wish to see republished in this country. We cannot but think, however, that the cases, making considerably more than half the volume, might be omitted, without materially diminishing the value of the book to the mass of the profession. But few American readers would study them, and as they double the price of the work, they could not fail to limit its circulation, an effect which we should sincerely deprecate. D.

